# The Clinical Characteristics of a Stage II Colorectal Cancer T4 Tumor: A Ten-Year Single-Center Research Report

**DOI:** 10.3390/curroncol31120584

**Published:** 2024-12-12

**Authors:** Bo-Zhi Lin, Chang-Lin Lin, Feng-Fan Chiang, Chou-Chen Chen, Ming-Cheng Chen, Chun-Yu Lin, Shang-Chih Huang

**Affiliations:** Taichung Veterans General Hospital, Taichung 407219, Taiwan; lpz1116@gmail.com (B.-Z.L.); hankel.chiang@gmail.com (F.-F.C.); ccchen407@yahoo.com (C.-C.C.); claudiochen7@gmail.com (M.-C.C.); classicpiano2003@gmail.com (C.-Y.L.); cheryl0036@gmail.com (S.-C.H.)

**Keywords:** stage II colorectal cancer, local recurrence, T4 tumor, adjuvant chemotherapy

## Abstract

Aim: The tumor staging of colorectal cancer (CRC) plays a significant role in both treatment and prognosis, impacting surgical planning and adjuvant therapy decisions. Currently, the staging of CRC is based on the TNM system developed by the American Joint Committee on Cancer. Prior studies have suggested that survival rates and recurrent rates of T4a tumors appear to be worse than that of T4b tumors, although there is currently no consensus. Therefore, we collected patient data from Taichung Veterans General Hospital over the past decade in order to conduct further research. Method: Between 2010 and 2018, a total of 5760 newly diagnosed CRC patients were seen at the hospital. To eliminate the influence of any local lymph node involvement or distant organ metastasis on the research results, we focused on patients with pathologic Stage IIc disease (T4a-bN0M0). Patients with rectal cancer who had received neoadjuvant concurrent chemoradiotherapy were excluded. Ultimately, 132 patients were included in this study. A multivariate Cox regression analysis was conducted to identify independent risk factors for both 10-year cancer-specific survival (CSS) and overall survival (OS). Results: A total of 132 patients were included in the study, with 90 classified as T4a and 42 as T4b. The 10-year CSS for pT4a and pT4b was 72.5% and 56.5%, respectively, with a *p*-value of 0.011. The 10-year OS for pT4a and pT4b was 48.4% and 42.5%, respectively, with a *p*-value of 0.086. There was no significant difference in the site of first recurrence between the pT4a and pT4b groups (*p*-value = 0.936). Overall, patients who received adjuvant chemotherapy therapy had a significantly better prognosis (*p*-value < 0.05). However, there was no significant difference in prognosis between oral 5-FU and FOLFOX. Conclusion: Based on our data, patients diagnosed with pathologic T4aN0M0 CRC appeared to experience a trend toward better 10-year OS when compared to those with T4bN0M0 disease, but this trend lacks statistical significance. Patients with locally advanced Stage II colon cancer clearly benefited from adjuvant chemotherapy therapy; therefore, FOLFOX may not necessarily be required.

## 1. Introduction

According to statistics from the Taiwan National Health Administration, the incidence and mortality rates of colorectal cancer (CRC) in Taiwan have been steadily increasing each year, with the disease consistently ranking as the leading form of cancer for the past 15 years. The treatment of CRC is staged according to the AJCC (American Joint Committee on Cancer) TNM system, with corresponding treatments recommended based upon the different stages. Patients with pathologic Stage I do not require adjuvant chemotherapy (ACT), while those who have reached Stage III are advised to undergo ACT. The treatment approach for Stage II patients, however, remains a topic of discussion [1]. Currently, decisions regarding the administration of chemotherapy are based on clinical and pathological risk assessments.

Since 2010, under the seventh edition of the AJCC staging system (AJCC-7) [2], T4 lesions have been further classified as either T4a (tumor penetrates the surface of the visceral peritoneum) or T4b (tumor directly invades or is histologically adherent to other organs or structures). Studies have confirmed that AJCC-7 has improved predictive capabilities for the prognosis of CRC [3]. There remains ongoing debate surrounding T4 colon tumors, as they are challenging to diagnose preoperatively using imaging, often presenting themselves with obstruction and perforation issues [4,5,6]. In 2020, an article suggested that neoadjuvant chemotherapy could be considered for clinical T4b stage colon cancer [7]; however, comprehensive treatment guidelines have not yet been fully established. Numerous studies have explored the management of T4 colon tumors. A 2019 article highlighted that, irrespective of the N stage, the pathological T4a (pT4a) stage is an independent risk factor for poorer oncologic outcomes compared to the pathological T3 (pT3) and T4b (pT4b) stages [8]. Additionally, pT4a tumors exhibit higher rates of local recurrence and peritoneal carcinomatosis. We are curious about the long-term prognosis and clinical outcomes of patients diagnosed with CRC when the postoperative pathology reveals a T4 tumor but no lymph node and the existence of distal organ metastasis. Up to now, there have been few articles in Taiwan discussing the differences between Stage II CRC involving pT4a and pT4b tumors. The objective of this study is to observe the long-term prognosis and other clinically relevant outcomes of Stage II T4 tumor patients, while also investigating the potential differences between the T4a and T4b stages. In addition to examining differences in survival rates, we aim to explore the benefits of ACT for this patient group and identify unique characteristics of disease recurrence in these cases.

## 2. Methods

### 2.1. Patients

This study is a retrospective analysis of a prospective database created by CRC case managers at Taichung Veterans General Hospital, beginning in 2010. The inclusion criteria encompassed all newly diagnosed and curatively operated CRC patients from the years 2010 to 2018, with postoperative pathology reports indicating T4a-bN0 and no clinical evidence of distal metastasis being reported. Patients receiving preoperative neoadjuvant therapy were excluded, as well as those with synchronous double primary cancer, postoperative complications leading to death, a history of previous malignancy, incomplete medical records, and patients who did not return for postoperative follow-up. Patients with R2 resection (macroscopic residual tumor peri-operation) were also excluded. A total of 132 patients were included in this study.

Data were collected on patient characteristics, including age, gender, body mass index (BMI), body surface area (BSA), Eastern Cooperative Oncology Group (ECOG) performance status, primary tumor location, high-risk pathological features (poor differentiation, perineural invasion, fewer than 12 lymph nodes harvested, lymphovascular invasion, positive resection margin), and high-risk clinical features (colonic obstruction, colonic perforation), as well as whether the patient received ACT. Dates of disease progression and recurrence sites were documented based on formal radiology reports. The surgical resection margin was defined as having a minimum of a 1 mm tumor-free margin. Tumor recurrence sites, including the liver, lung, peritoneum, and local recurrence, were recorded based on imaging and colonoscopy findings.

Patients who received ACT were divided into two groups: oral 5-FU (5-Fluorouracil) and standard mFOLFOX6 (5-Fluorouracil, leucovorin and oxaliplatin) treatment. Prior to 2018, our hospital did not undergo the practice of using the regimen of Oral 5-FU + Oxaliplatin. Therefore, in our hospital, the decision to administer ACT was based on the clinical physician’s discretion or shared decision-making with the patient.

The management of patients following disease recurrence was categorized into three groups: best supportive care (BSC), operation ± systemic treatment, and systemic treatment only. All post-recurrence treatment decisions were determined through a Multidisciplinary Team meeting. Patients included in the operation group were those preoperatively assessed as candidates for curative resection. Surgeries performed solely to address symptoms (e.g., diverting colostomy) were classified under the BSC group.

### 2.2. Statistical Analysis

Data were retrospectively collected from the hospital’s database. Continuous variables are presented as medians and were compared using the Mann–Whitney U test. Categorical variables are expressed as frequencies and percentages and were analyzed using Chi-square tests. Survival curves were estimated using the Kaplan–Meier method. Overall survival (OS) and cancer-specific survival (CSS) were compared between the pT4a and pT4b groups using the log-rank test. Additionally, a multivariate Cox regression analysis with a stepwise procedure was conducted to identify independent risk factors for 10-year CSS, progression-free survival (PFS), and OS.

The rationale for selecting OS and CSS as 10-year prognostic indicators lies in the extended follow-up period, which enables a comprehensive assessment of patient survival by distinguishing between cancer-related and non-cancer-related causes of death. Over a 10-year follow-up, most patients are likely to have experienced disease progression, thereby diminishing the relevance of progression-free survival (PFS) data in the later stages. However, we also used the log-rank test to compare 10-year PFS differences between the pT4a group and the pT4b group, with the corresponding figures included in the Supplementary Materials.

In the subgroup analysis, we also used the Kaplan–Meier method to construct survival curves, comparing OS differences between the oral 5-FU group and the standard mFOLFOX6 group, as well as differences in OS within the tumor recurrence group. All statistical analyses were conducted using Statistical Product and Service Solutions (SPSS), version 22 (IBM Corp., Armonk, NY, USA).

## 3. Results

**Figure 1:**
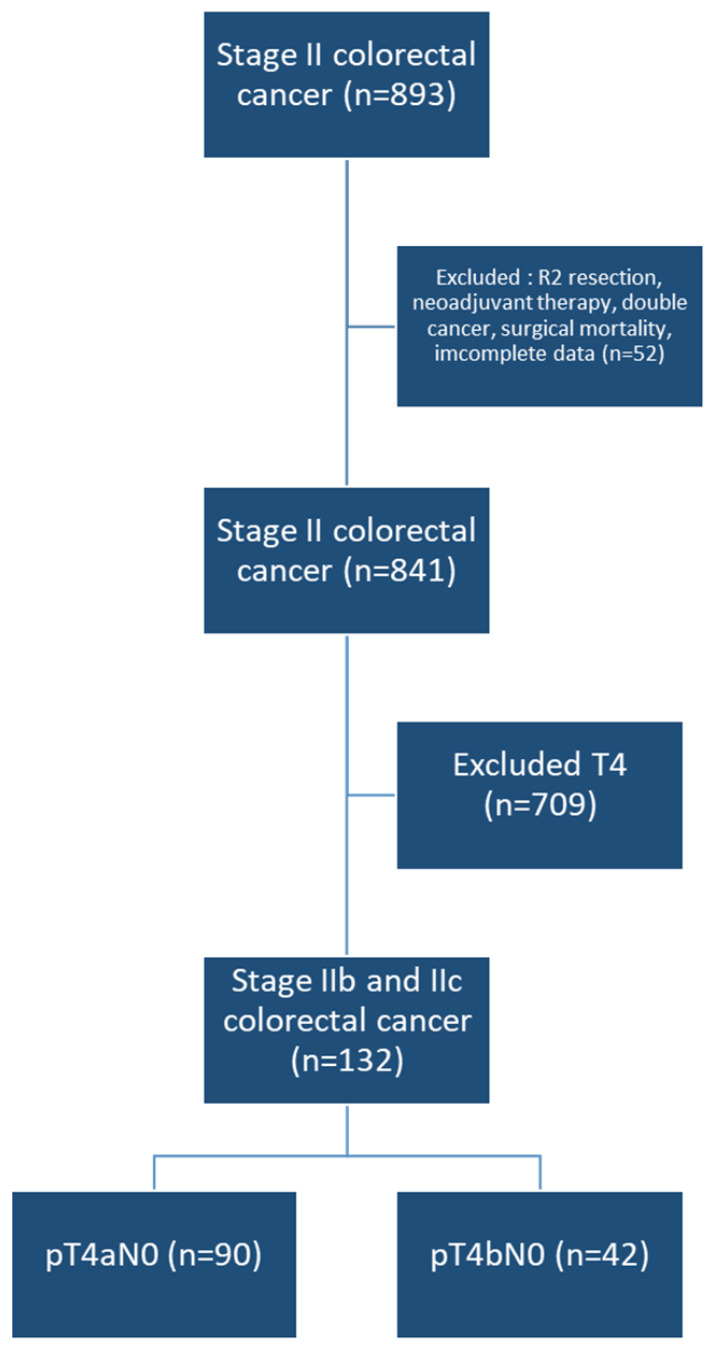
Flow chart of patients selection.

A total of 132 patients were included in the study, with 90 classified as T4a and 42 as T4b (Figure 1). There were no differences between the two groups in terms of average age, gender distribution, primary tumor site (left or right colon) or clinicopathological risk features. However, patients in the T4a group had a higher BMI and a lower ECOG PS Scale (Table 1).

**Figure 2:**
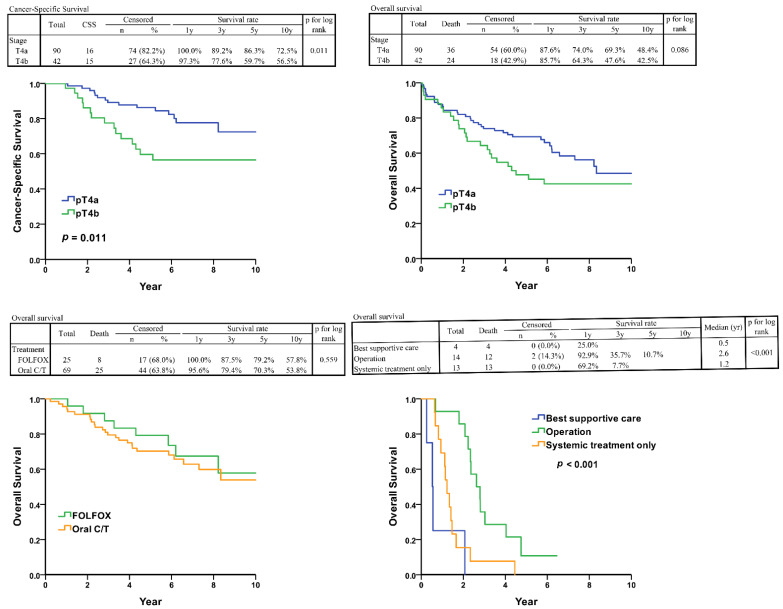
(**Upper left**): Cancer-specific survival of Stage II T4 colorectal cancer. (**Upper right**): Overall-survival survival of Stage II T4 colorectal cancer. (**Lower left**): Overall survival of adjuvant therapy group. (**Lower right**): Overall survival of tumor recurrent group. C/T: chemotherapy, specifically the use of oral 5-FU medications.

The 10-year CSS rates for pT4a and pT4b were 72.5% and 56.5%, respectively, with a *p*-value of 0.011 (Figure 2). The 10-year OS rates for pT4a and pT4b were 48.4% and 42.5%, respectively, with a *p*-value of 0.086 (Figure 2). In the multivariate Cox regression analysis, age, BMI, and receipt of adjuvant chemotherapy (ACT) were significant predictors of OS (*p* < 0.05; Table 2). For CSS, only poor differentiation and pT4b were associated with significantly worse outcomes (*p* < 0.05; Table 3). Finally, regarding PFS, older age, lower BMI, absence of ACT, and poor differentiation were significantly associated with shorter PFS (*p* < 0.05; Table 4). However, there was no significant difference in prognosis between patients receiving oral 5-FU and those receiving intravenous FOLFOX (*p*-value = 0.559) (Figure 2).

A total of 31 patients experienced disease recurrence, with recurrence rates of 3.8% (5/132) in the liver, 3.8% (5/132) in the lungs, 7.6% (10/132) for local recurrence, and 8.3% (11/132) in the peritoneum. The distribution of recurrence sites between the pT4a and pT4b groups was nearly identical (*p* = 0.928). (Table 5). Among these 31 patients, those who received BSC after recurrence had a median survival of 0.6 years. Patients who underwent at least an R1 resection (no residual macroscopic tumor, but microscopic margins which still demonstrated the presence of tumor) +/− chemotherapy had a median survival of 2.6 years, while patients who did not undergo surgery but received salvage chemotherapy had a median survival of 1.2 years, with statistically significant differences (*p*-value < 0.001) (Figure 2).

## 4. Discussion

For Stage II CRC, the decision to undergo adjuvant therapy postoperatively hinges upon a thorough risk assessment. As the old adage suggests, “A word before is worth two behind.” Clinical practitioners are dedicated to deploying a myriad of methods in order to precisely predict the risks associated with Stage II CRC. Traditionally, these predictions have been based on clinical and pathological features [9,10,11]. However, as our understanding better evolves over time, additional concerns such as tumor budding [12,13,14] and the concentration of ctDNA (circulating tumor DNA) measured by NGS (next-generation sequencing) have all gained significance in the risk assessment process [15].

The challenges intensify when dealing with T4 tumors, which indicates the invasion of the visceral peritoneum. T4b tumors often necessitate the resection of adjacent organs, leading to disruptions in planned surgical procedures, while T4a tumors are associated with an elevated risk of carcinomatosis, a concern highlighted in the previous literature [16]. It is important to note that pathological T4 tumors are considered high-risk factors for recurrence, as emphasized in the NCCN guidelines [17].

Our data analysis unveiled compelling insights, particularly regarding the impact of poor differentiation on the long-term prognosis of patients diagnosed with pathological T4 tumors. Notably, within this cohort, 22.7% (30/132) experienced colonic obstruction, while 8.3% (11/132) faced complications of perforation. Additionally, 15.9% (21/132) of patients exhibited positive surgical margins. Intriguingly, these three findings did not exert a significant influence on either cancer-specific survival (CSS) or overall survival (OS). Based on prior studies, perforation and obstruction in CRC have been associated with poorer long-term prognosis, reducing three-year progression-free survival (PFS) by over 35% [18]. Another study focusing on Stage II-perforated CRC demonstrated a 23% increased risk of recurrence due to perforation [19]. Both studies highlighted that T4 tumors are inherently more prone to causing obstruction or perforation. In our study, however, the observed cohort was strictly limited to Stage II T4 CRC patients, which we believe explains these results. The homogeneous patient population may have reduced variability and diminished the impact of these complications on long-term outcomes. Regarding positive surgical margins, it is important to note that radial margin positivity in colon cancer is relatively uncommon. A retrospective study reported an incidence of only 5.3%, which was associated with poorer long-term prognosis [20]. However, the higher proportion of positive surgical margins in our study is reasonable given that we included only T4 tumors, which are more prone to margin positivity. We attribute the lack of impact from positive surgical margins on long-term outcomes to the fact that all patients in our study underwent gross complete resection (R1 resection), with more than 80% (17/21) receiving postoperative adjuvant therapy. This comprehensive approach of complete resection combined with adjuvant therapy may mitigate the adverse effects of positive surgical margins on long-term outcomes.

Furthermore, our investigation identified a significant correlation between BMI and long-term prognosis (OS, *p* = 0.036, HR = 0.91). Notably, this finding revealed a protective effect of higher BMI on the prognosis of Stage II CRC patients with pathological T4 tumors. This result aligns with and reinforces previous research in the field, emphasizing BMI’s important role in influencing the course of CRC. A retrospective study involving 3799 patients indicated that overweight individuals with Stage I and Stage II CRC exhibited a trend toward better survival, although this did not reach statistical significance [21]. Additionally, a meta-analysis revealed that underweight patients have poorer long-term outcomes, whereas those classified as overweight (BMI 25–30) demonstrated improved survival [22]. The protective effect of a higher BMI underscores a potential avenue for further investigation, particularly in developing tailored interventions to enhance outcomes for patients with specific tumor characteristics.

Examining the 10-year OS and CSS, the OS rate for pT4aN0 was 48.4%, while the CSS rate was 72.5%. For pT4bN0, the OS rate was 42.5%, and the CSS rate was 56.5%. The Kaplan–Meier survival curve indicated a slightly prolonged trend for pT4a compared to pT4b. However, a univariable Cox analysis revealed no statistically significant difference between the two groups in terms of OS (HR: 1.57, *p* = 0.089). A study published in 2024, which similarly used a retrospective cohort of pT4N0 patients, included 184 cases. The findings regarding OS were comparable to ours: the median OS for pT4a was 115.1 months, and for pT4b, it was 99.2 months. Their univariable analysis also showed no statistically significant difference (*p* = 0.541) [23]. However, due to our longer follow-up period, we also utilized CSS as a prognostic metric, which they did not. Our results demonstrated a significant difference in CSS, highlighting the added value of using CSS for long-term survival evaluation. Additionally, another article published in May 2024, focusing on Stage II CRC with T4 tumors [24], reported that pT4a tumors exhibited significantly better 3-year OS (*p* = 0.001). This finding contradicts earlier studies that identified pT4a as an independent risk factor [25], at least within the context of Stage II CRC.

The overall recurrence rate in our cohort was approximately 23.5% (31/132), with a higher proportion of peritoneal metastasis and local recurrence compared to distal organ metastasis. A 2018 study involving 208 high-risk Stage II CRC patients reported an overall recurrence rate of 13%, with distal organ metastasis being more common than peritoneal metastasis and local recurrence [26]. This suggests that T4 tumors may be more prone to peritoneal metastasis and local recurrence following surgery. CRC recurrence is relatively common, and aggressive surgical intervention can still benefit select patients [27], even when the recurrence is distal or local. A 2020 review article reported that patients with locally recurrent colon cancer who underwent radical salvage surgery achieved a 5-year overall survival (OS) rate of 27–46% [28]. Even for patients with peritoneal metastasis at recurrence, cytoreductive surgery followed by adjuvant chemotherapy (ACT) resulted in a 5-year OS rate exceeding 25%. However, the benefit of surgery diminishes when the peritoneal carcinomatosis index exceeds 15, as no significant advantage has been demonstrated in such cases. Among our 14 surgical patients, 2 (14.3%) achieved complete disease remission, with no detectable recurrence beyond five years post-surgery. These findings highlight the importance of recommending aggressive treatment strategies for patients with pT4 CRC who exhibit the potential for resectable disease recurrence.

T4 tumors in Stage II CRC have long been considered a high-risk factor for disease recurrence, and its outcome in patients is similar to T3N1 CRC [29]. The administration of adjuvant therapy to patients diagnosed with Stage II local advanced CRC is universally undisputed. Previous articles have argued that not all risk factors have the same impact, and T4 tumors, in fact, benefit more from adjuvant therapy [30]. In our data, although there was no statistically significant difference in CSS with the administration of adjuvant therapy, PFS showed a significant improvement with ACT. We believe that PFS is more sensitive to interventions that impact disease progression. Since adjuvant chemotherapy effectively delays progression, its benefits are more readily observed in PFS. In Stage II CRC, the standard options are oral 5-FU or intravenous FOLFOX [31]. Our data suggest that there is no significant difference in prognosis between the two treatment options. Considering the potential for oxaliplatin to cause residual neurotoxic side effects, it may be reasonable to consider either intravenous infusions of 5-FU or oral 5-FU alone.

## 5. Limitations

There are several limitations to our study. Firstly, as a single-center retrospective study, it is inherently subject to selection bias and confounding variables. Although a multidisciplinary team was involved in planning cancer treatments, variations in individual preferences among treating physicians may still have influenced decisions. While two cancer case managers were responsible for tracking patients, the extended follow-up period of over a decade may have resulted in inconsistencies in the timing and quality of data collection, potentially affecting the accuracy and reliability of the results. Furthermore, the inclusion of only Stage IIb–IIc patients led to a relatively small sample size, which may limit the statistical power and precision of our findings.

## 6. Conclusions

In summary, patients diagnosed with Stage II pT4a CRC show a better long-term prognosis compared to those with pT4b. The location of tumor recurrence did not differ significantly between the two groups. ACT, whether using oral 5-FU alone or the FOLFOX regimen, provides significant benefits for these patients.

## Figures and Tables

**Table 1:** Baseline characteristics of patients with Stage II pT4 colon cancer (N = 132).

	T4a		T4b		*p*-Value
N	90		42		
Age	67.0	(55.0–75.0)	57.5	(52.8–72.3)	0.096
Gender					0.742
Female	37	(41.1%)	16	(38.1%)	
Male	53	(58.9%)	26	(61.9%)	
BMI	22.9	(21.1–25.0)	21.3	(19.2–24.8)	0.039 *
BSA	1.6	(1.5–1.8)	1.6	(1.5–1.8)	0.942
ECOG					0.025 *
0	12	(13.3%)	1	(2.4%)	
1	48	(53.3%)	18	(42.9%)	
2–3	30	(33.3%)	23	(54.8%)	
Site					0.421
Left	58	(64.4%)	24	(57.1%)	
Right	32	(35.6%)	18	(42.9%)	
ACT					0.043 *
No	21	(23.3%)	17	(40.5%)	
Yes	69	(76.7%)	25	(59.5%)	
Poor diffierential					0.725
No	84	(93.3%)	38	(90.5%)	
Yes	6	(6.7%)	4	(9.5%)	
Lympho/vascular invasion					0.882
No	78	(86.7%)	36	(85.7%)	
Yes	12	(13.3%)	6	(14.3%)	
Obstruction					0.256
No	67	(74.4%)	35	(83.3%)	
Yes	23	(25.6%)	7	(16.7%)	
<12 lymph nodes examined					--
No	90	(100.0%)	42	(100.0%)	
Yes	0	(0.0%)	0	(0.0%)	
Perineural invasion					0.062
No	61	(67.8%)	35	(83.3%)	
Yes	29	(32.2%)	7	(16.7%)	
Perforation					0.743
No	83	(92.2%)	38	(90.5%)	
Yes	7	(7.8%)	4	(9.5%)	
Margins positive					0.390
No	74	(82.2%)	37	(88.1%)	
Yes	16	(17.8%)	5	(11.9%)	

Chi-square test or Mann–Whitney U test. Median (IQR) * *p* < 0.05. BMI: body mass index, BSA: body surface area, ECOG: Eastern Cooperative Oncology Group, ACT: adjuvant chemotherapy.

**Table 2:** Cox regression—overall survival.

	Univariate			Multivariable (Stepwise)		
	Hazard Ratio	95% CI	*p*-Value	Hazard Ratio	95% CI	*p*-Value
Age	1.05	(1.03–1.07)	<0.001 **	1.04	(1.02–1.06)	<0.001 **
Gender						
Female	Reference					
Male	1.56	(0.91–2.67)	0.108			
BMI	0.92	(0.85–1.00)	0.053	0.91	(0.84–0.98)	0.012 *
ECOG						
0–1	Reference			Reference		
2–3	3.17	(1.89–5.32)	<0.001 **	2.71	(1.61–4.57)	<0.001 **
Site						
Left	Reference					
Right	1.67	(1.00–2.78)	0.048 *			
Adjuvant C/T						
No	Reference			Reference		
Yes	0.31	(0.19–0.52)	<0.001 **	0.44	(0.25–0.75)	0.003 **
Poor diffierential						
No	Reference					
Yes	1.86	(0.84–4.10)	0.123			
Lympho/vascular invasion						
No	Reference					
Yes	1.29	(0.67–2.50)	0.441			
Obstruction						
No	Reference					
Yes	1.66	(0.95–2.91)	0.078			
Perineural invasion						
No	Reference					
Yes	0.82	(0.45–1.49)	0.517			
Perforation						
No	Reference					
Yes	1.69	(0.72–3.93)	0.225			
Margins positive						
No	Reference					
Yes	0.76	(0.36–1.60)	0.468			
Stage						
T4a	Reference					
T4b	1.57	(0.93–2.63)	0.089			

Cox proportional hazard regression. * *p* < 0.05, ** *p* < 0.01. BMI: body mass index, BSA: body surface area, ECOG: Eastern Cooperative Oncology Group, ACT: adjuvant chemotherapy.

**Table 3:** Cox regression—cancer specific survival.

	Univariate			Multivariable (Stepwise)		
	Hazard Ratio	95% CI	*p*-Value	Hazard Ratio	95% CI	*p*-Value
Age	1.00	(0.97–1.03)	0.878			
Gender						
Female	Reference					
Male	1.51	(0.70–3.25)	0.297			
BMI	0.95	(0.85–1.06)	0.337			
ECOG						
0–1	Reference			Reference		
2–3	3.53	(1.68–7.40)	0.001 **	2.83	(1.32–6.09)	0.008 **
Site						
Left	Reference					
Right	1.64	(0.79–3.41)	0.187			
Adjuvant C/T						
No	Reference					
Yes	0.51	(0.23–1.12)	0.094			
Poor diffierential						
No	Reference			Reference		
Yes	3.07	(1.17–8.06)	0.023 *	3.55	(1.28–9.81)	0.015 *
Lympho/vascular invasion						
No	Reference					
Yes	1.52	(0.61–3.74)	0.366			
Obstruction						
No	Reference					
Yes	0.92	(0.35–2.41)	0.861			
Perineural invasion						
No	Reference					
Yes	0.71	(0.29–1.74)	0.449			
Perforation						
No	Reference					
Yes	1.96	(0.59–6.48)	0.272			
Margins positive						
No	Reference					
Yes	0.75	(0.26–2.15)	0.585			
Stage						
T4a	Reference			Reference		
T4b	2.62	(1.26–5.43)	0.010 *	2.47	(1.14–5.36)	0.022 *

Cox proportional hazard regression. * *p* < 0.05, ** *p* < 0.01. BMI: body mass index, BSA: body surface area, ECOG: Eastern Cooperative Oncology Group, ACT: adjuvant chemotherapy.

**Table 4:** Cox regression—progression-free survival.

	Univariate			Multivariable (Stepwise)		
	Hazard Ratio	95%CI	*p*-Value	Hazard Ratio	95%CI	*p*-Value
Age	1.04	(1.02–1.06)	<0.001 **	1.03	(1.01–1.05)	0.012 *
Gender						
Female	Reference					
Male	1.43	(0.84–2.42)	0.186			
BMI	0.92	(0.85–1.00)	0.039 *	0.92	(0.85–0.99)	0.026 *
ECOG						
0–1	Reference			Reference		
2–3	3.01	(1.81–4.98)	<0.001 **	2.47	(1.47–4.14)	0.001 **
Site						
Left	Reference					
Right	1.58	(0.96–2.61)	0.072			
Adjuvant C/T						
No	Reference			Reference		
Yes	0.33	(0.20–0.55)	<0.001 **	0.44	(0.25–0.76)	0.003 **
Poor diffierential						
No	Reference			Reference		
Yes	2.31	(1.09–4.86)	0.028 *	2.57	(1.19–5.57)	0.017 *
Lympho/vascular invasion						
No	Reference					
Yes	1.31	(0.68–2.52)	0.419			
Obstruction						
No	Reference					
Yes	1.54	(0.88–2.69)	0.130			
Perineural invasion						
No	Reference					
Yes	0.85	(0.48–1.53)	0.595			
Perforation						
No	Reference					
Yes	1.59	(0.68–3.70)	0.282			
Margins positive						
No	Reference					
Yes	0.76	(0.36–1.59)	0.459			
Stage						
T4a	Reference					
T4b	1.51	(0.90–2.51)	0.117			

Cox proportional hazard regression. * *p* < 0.05, ** *p* < 0.01. BMI: body mass index, BSA: body surface area, ECOG: Eastern Cooperative Oncology Group, ACT: adjuvant chemotherapy.

**Table 5:** Distribution of recurrent sites.

	pT4a		pT4b		*p*-Value
Recurrent site					0.928
Liver	3	(18.8%)	2	(13.3%)	
Lung	3	(18.8%)	2	(13.3%)	
Local recurrence	5	(31.3%)	5	(33.3%)	
Peritoneum	5	(31.3%)	6	(40.0%)	

Chi-square test.

## Data Availability

Data can be made available from the corresponding author on reasonable request.

## Supplementary Materials

The following supporting information can be downloaded at https://www.mdpi.com/article/10.3390/curroncol31120584/s1: Figure S1: Progression free survival between T4a and T4b.

## References

[1] André T., Boni C., Navarro M., Tabernero J., Hickish T., Topham C., Bonetti A., Clingan P., Bridgewater J., Rivera F. (2009). Improved overall survival with oxaliplatin, fluorouracil, and leucovorin as adjuvant treatment in stage II or III colon cancer in the MOSAIC trial. J. Clin. Oncol..

[2] Edge S.B., Compton C.C. (2010). The American Joint Committee on Cancer: The 7th Edition of the AJCC Cancer Staging Manual and the Future of TNM. Ann. Surg. Oncol..

[3] Gao P., Song Y.X., Wang Z.N., Xu Y.Y., Tong L.L., Sun J.X., Yu M., Xu H.M. (2013). Is the prediction of prognosis not improved by the seventh edition of the TNM classification for colorectal cancer? Analysis of the surveillance, epidemiology, and end results (SEER) database. BMC Cancer.

[4] Helewa R.M., Park J. (2016). Surgery for Locally Advanced T4 Rectal Cancer: Strategies and Techniques. Clin. Colon. Rectal Surg..

[5] Gosavi R., Heriot A.G., Warrier S.K. (2020). Current management and controversies in management of T4 cancers of the colon—A narrative review of the literature. Dig. Med. Res..

[6] El-Sharkawy F., Gushchin V., Plerhoples T.A., Liu C., Emery E.L., Collins D.T., Bijelic L. (2021). Minimally invasive surgery for T4 colon cancer is associated with better outcomes compared to open surgery in the National Cancer Database. Eur. J. Surg. Oncol..

[7] Gosavi R., Chia C., Michael M., Heriot A.G., Warrier S.K., Kong J.C. (2021). Neoadjuvant chemotherapy in locally advanced colon cancer: A systematic review and meta-analysis. Int. J. Colorectal Dis..

[8] Baguena G., Pellino G., Frasson M., Roselló S., Cervantes A., García-Granero A., Giner F., García-Granero E. (2019). Prognostic Impact of pT Stage and Peritoneal Invasion in Locally Advanced Colon Cancer. Dis. Colon. Rectum.

[9] Artac M., Turhal N.S., Kocer M., Karabulut B., Bozcuk H., Yalcin S., Karaagac M., Gündüz S., Isik N., Uygun K. (2014). Do high-risk features support the use of adjuvant chemotherapy in stage II colon cancer? A Turkish Oncology Group study. Tumori.

[10] Kumar A., Kennecke H.F., Renouf D.J., Lim H.J., Gill S., Woods R., Speers C., Cheung W.Y. (2015). Adjuvant chemotherapy use and outcomes of patients with high-risk versus low-risk stage II colon cancer. Cancer.

[11] Casadaban L., Rauscher G., Aklilu M., Villenes D., Freels S., Maker A.V. (2016). Adjuvant chemotherapy is associated with improved survival in patients with stage II colon cancer. Cancer.

[12] Petrelli F., Pezzica E., Cabiddu M., Coinu A., Borgonovo K., Ghilardi M., Lonati V., Corti D., Barni S. (2015). Tumour Budding and Survival in Stage II Colorectal Cancer: A Systematic Review and Pooled Analysis. J. Gastrointest. Cancer.

[13] Zlobec I., Berger M.D., Lugli A. (2020). Tumour budding and its clinical implications in gastrointestinal cancers. Br. J. Cancer.

[14] Saito K., Okuyama T., Miyazaki S., Oi H., Mitsui T., Noro T., Takeshita E., Ono Y., Urahashi T., Tajima H. (2022). Tumor Budding as a Predictive Marker of Relapse and Survival in Patients With Stage II Colon Cancer. In Vivo.

[15] Tie J., Cohen J.D., Lahouel K., Lo S.N., Wang Y., Kosmider S., Wong R., Shapiro J., Lee M., Harris S. (2022). Circulating Tumor DNA Analysis Guiding Adjuvant Therapy in Stage II Colon Cancer. N. Engl. J. Med..

[16] Hompes D., Tiek J., Wolthuis A., Fieuws S., Penninckx F., Van Cutsem E., D’Hoore A. (2012). HIPEC in T4a colon cancer: A defendable treatment to improve oncologic outcome?. Ann. Oncol..

[17] Benson A.B., Venook A.P., Al-Hawary M.M., Arain M.A., Chen Y.J., Ciombor K.K., Cohen S., Cooper H.S., Deming D., Farkas L. (2021). Colon Cancer, Version 2.2021, NCCN Clinical Practice Guidelines in Oncology. J. Natl. Compr. Canc Netw..

[18] Yang K.M., Jeong M.J., Yoon K.H., Jung Y.T., Kwak J.Y. (2022). Oncologic outcome of colon cancer with perforation and obstruction. BMC Gastroenterol..

[19] Asano H., Fukano H., Takagi M., Takayama T. (2023). Risk factors for the recurrence of stage II perforated colorectal cancer: A retrospective observational study. Asian J. Surg..

[20] Amri R., Bordeianou L.G., Sylla P., Berger D.L. (2015). Association of Radial Margin Positivity With Colon Cancer. JAMA Surgery.

[21] Shahjehan F., Merchea A., Cochuyt J.J., Li Z., Colibaseanu D.T., Kasi P.M. (2018). Body Mass Index and Long-Term Outcomes in Patients With Colorectal Cancer. Front. Oncol..

[22] Simillis C., Taylor B., Ahmad A., Lal N., Afxentiou T., Powar M.P., Smyth E.C., Fearnhead N.S., Wheeler J., Davies R.J. (2022). A systematic review and meta-analysis assessing the impact of body mass index on long-term survival outcomes after surgery for colorectal cancer. Eur. J. Cancer.

[23] Akdag G., Isik D., Dogan A., Yildirim S., Kinikoglu O., Topal A., Oksuz S., Turkoglu E., Surmeli H., Basoglu T. (2024). Does Adjuvant Chemotherapy Benefit Patients with T4 N0 Colon Cancer?. Medicina.

[24] Liu B., Zhang Z.X., Nie X.Y., Sun W.L., Yan Y.J., Fu W.H. (2024). Clinical outcome and prognostic factors of T4N0M0 colon cancer after R0 resection: A retrospective study. World J. Gastrointest. Oncol..

[25] Kumamoto T., Yamaguchi S., Nakagawa R., Nagashima Y., Maeda F., Tani K., Kondo H., Koshino K., Kaneko Y., Bamba Y. (2023). Prognostic risk factors for pT4 colon cancer: A retrospective cohort study. Oncol. Lett..

[26] Nozawa H., Kawai K., Hata K., Tanaka T., Nishikawa T., Otani K., Sasaki K., Kaneko M., Emoto S., Murono K. (2018). High-risk Stage II Colorectal Cancers Carry an Equivalent Risk of Peritoneal Recurrence to Stage III. In Vivo.

[27] Hellinger M.D., Santiago C.A. (2006). Reoperation for recurrent colorectal cancer. Clin. Colon. Rectal Surg..

[28] Brown K.G.M., Koh C.E. (2020). Surgical management of recurrent colon cancer. J. Gastrointest. Oncol..

[29] Chan D.K.H., Lim T.Z., Tan K.K. (2019). T4N0 colon cancers should be treated like T3N1 disease. J. Gastrointest. Oncol..

[30] Babcock B.D., Aljehani M.A., Jabo B., Choi A.H., Morgan J.W., Selleck M.J., Luca F., Raskin E., Reeves M.E., Garberoglio C.A. (2018). High-Risk Stage II Colon Cancer: Not All Risks Are Created Equal. Ann. Surg. Oncol..

[31] Varghese A. (2015). Chemotherapy for Stage II Colon Cancer. Clin. Colon. Rectal Surg..

