# Supplementary figures and images for: The Clinical Characteristics of a Stage II Colorectal Cancer T4 Tumor: A Ten-Year Single-Center Research Report
Source: Curr Oncol. 2024 Dec 12;31(12):7924–35. doi: 10.3390/curroncol31120584 (PMC11674692; doi:10.3390/curroncol31120584)

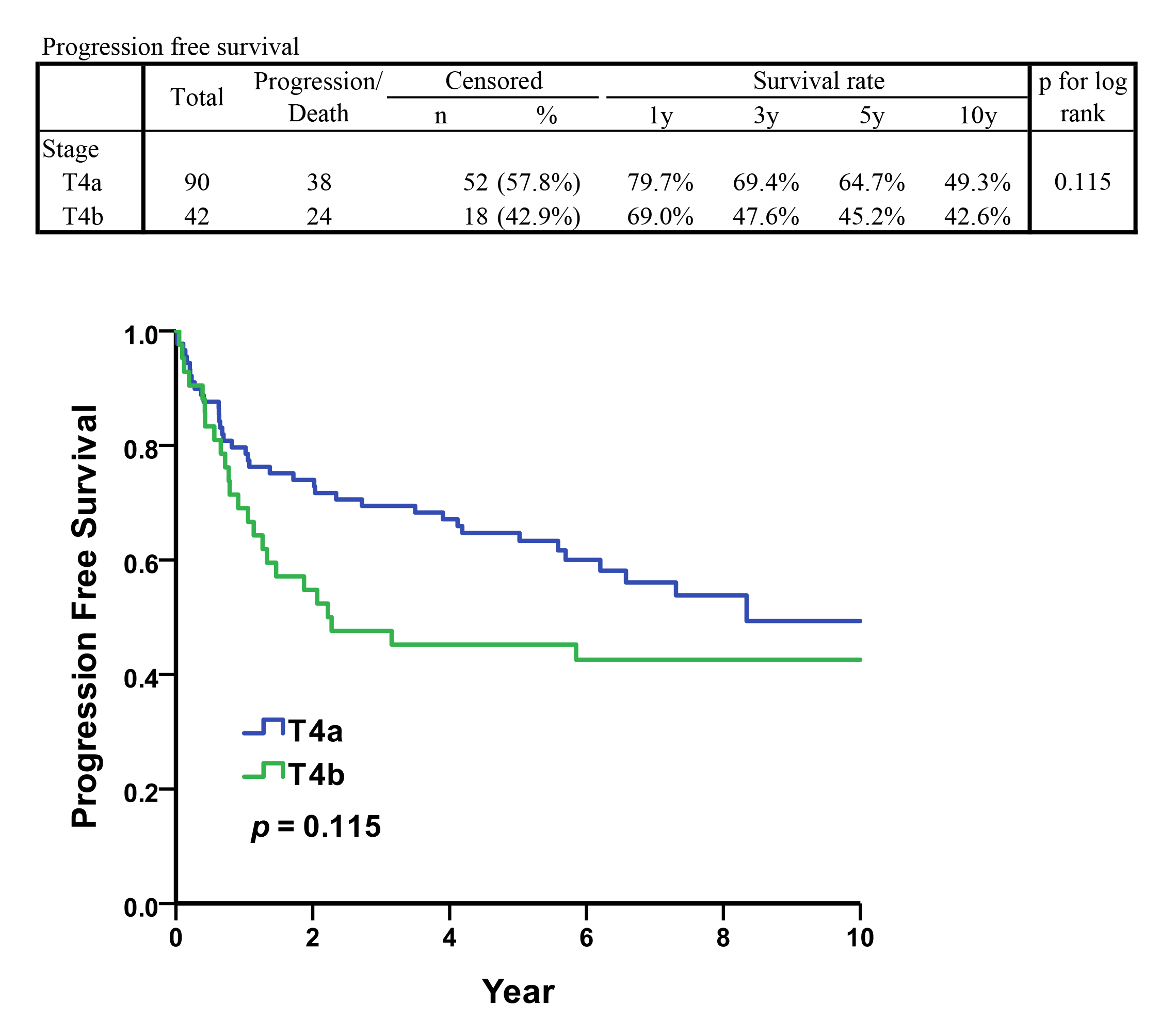

Supplement: Supplementary file 1 [file curroncol-31-00584-s001.zip › curroncol-3337214-supplementary.tif]
